# Regarding: ‘identification of risk factors and development of predictive models for chronic postsurgical pain after modified radical mastectomy’

**DOI:** 10.1080/07853890.2026.2637252

**Published:** 2026-03-07

**Authors:** Qiuhua Zhang, Huimin Jin

**Affiliations:** a The First School of Clinical Medicine, Zhejiang Chinese Medical University, Hangzhou, China; b Department of Oncology, The Second Affiliated Hospital of Zhejiang Chinese Medical University, Hangzhou, China

Dear Editor

We read with great interest the article by Gong et al. [1] titled ‘Identification of risk factors and development of predictive models for chronic postsurgical pain after modified radical mastectomy’. The authors conducted a prospective cohort study to identify predictors of chronic postsurgical pain (CPSP) at 3 and 6 months postoperatively and developed nomogram-based prediction models. We commend the authors for addressing an important clinical issue with a well-structured study design, we would like to highlight several methodological and interpretative concerns that merit further consideration.

It is worth noting that CPSP was defined in this study as an NRS score > 0, encompassing mild pain. While the authors have highlighted the adverse impact of mild persistent pain on patients’ quality of life, this definition may overestimate the actual clinical burden of CPSP and potentially attenuate the strength of associations between core predictors and clinically significant moderate-to-severe pain. It is therefore recommended that future studies employ more stringent pain thresholds (e.g. NRS ≥ 3) or integrate pain phenotypes (a limitation noted in the discussion section of the original article) for stratified analyses. Such an approach would further improve the clinical applicability of the prediction model and enhance the validity of result interpretation.

While the authors adhered to the traditional principle of EPV ≥ 10 for sample size estimation, modern prediction model methodologies favor adopting a more conservative threshold of EPV ≥ 15–20 to enhance model robustness [2,3]. The actual EPV values of the final models were approximately 23.8 for the 3-month cohort and 18.2 for the 6-month cohort, with the 6-month model approaching the lower bound of the conservative standard. Furthermore, the study employed a data-driven variable selection strategy(preliminary univariate screening combined with LASSO regression) and explored nonlinear relationships *via* restricted cubic spline (RCS) models. Such complex analytical procedures may significantly elevate the risk of overfitting given the limited sample size. Although 10-fold cross-validation demonstrated the model’s internal stability,key performance metrics such as discriminative ability and calibration in independent external populations still require further verification through rigorous multicenter external validation.

Finally, while the study innovatively highlights FSH as a predictive factor for CPSP, however, the rationale for not conducting multivariate analysis or mechanistic exploration of other measured sex hormones—such as estrogen, progesterone, and testosterone—remains inadequately explained. The criteria for variable exclusion, such as detailed collinearity diagnostics or quantitative evidence of non-association, were not explicitly provided. Moreover,the omission of key covariates strongly linked to CPSP—such as pre-operative pain sensitivity and the fine details of intra-operative nerve management—may confound the accurate estimation of the independent effects of core predictors like FSH and postoperative insomnia, thereby limiting a deeper understanding of the mechanisms underlying CPSP development.

Gong et al. have made a valuable insights into the multifactorial etiology of CPSP after breast cancer surgery. Their findings underscore the roles of endocrine and sleep-related factors in chronic pain development. With further validation and refinement, their predictive models could inform personalized prevention strategies in clinical practice.

## Data Availability

Data sharing is not applicable to this article as no new data were created or analysed in this research.
